# Are Shiftwork and Indoor Work Related to D3 Vitamin Deficiency? A Systematic Review of Current Evidences

**DOI:** 10.1155/2018/8468742

**Published:** 2018-09-10

**Authors:** Luca Coppeta, Francesca Papa, Andrea Magrini

**Affiliations:** Department of Occupational Medicine, University of Rome “Tor Vergata”, Rome, Italy

## Abstract

**Background:**

Reported cases of vitamin D3 deficiency have been increasing in incidence worldwide. Although there is a lack of consensus relating to optimal levels of vitamin D, generally serum 25-(OH)D concentrations lower than 50 nmol/L (20 ng/mL) are at least considered to be detrimental to bone health.

**Aim:**

Aim of this systematic review is to investigate if occupations, and specifically shiftworking and indoor working, may be considered as possible contributors to the increased incidence of vitamin D3 deficiency in industrialized nations.

**Materials and Methods:**

Systematic review was performed according to the Preferred Reporting Items for Systematic Reviews and Meta-Analyses (PRISMA) statement using PubMed, Scopus, and ISI Web of Knowledge databases.

**Results:**

Overall 90 papers were found, 23 articles through PubMed, 30 through Scopus, and 37 through ISI Web of Knowledge. Successively, 46 duplicates and 34 articles that did not respect the inclusion criteria were excluded. Finally 10 articles were selected: 9 cross-sectional studies and 1 systematic review. Results of the studies included revealed that certain occupations are either suffering from, or have a predilection to suffer from, a deficiency of this vitamin. Shiftworkers and indoor workers are consistently reported as being the occupational group most likely to suffer from a deficiency of vitamin D3. It would appear prudent to investigate the potential of providing nutritional education to workers in addition to including preventative measures in the workplace.

## 1. Introduction

Reported cases of vitamin D3 deficiency have been increasing in incidence and now reach worldwide prevalence even in countries where sunlight exposure is high [1, 2]. The implications are vast not only in respect of health consequences and associated costs but also to economies in general as increasing numbers of the working population are affected. It is now suggested that around 40% of the US population are vitamin D deficient [3] while the figures provided for Canada suggest the proportion affected is around 32% depending on demographic and season [4]. Although vitamin D3 deficiency is commonly associated with issues such as bone health, it has also been linked with many other illnesses including autoimmune conditions, metabolic function, some cancers, and also psychiatric disorders [3, 5, 6].

Vitamin D3 differs from other vitamins not only in that it is a hormone but also because it is not uniquely obtained from certain foods by ingestion. The greatest proportion of vitamin D, around 90%, is synthesized by exposure of skin to sunlight [3]. This endogenous production is achieved by in vivo synthesis when ultraviolet B (UVB) light comes into contact with the skin and interacts with the precursor molecule 7-dehydrocholesterol. Conversion of vitamin D to 25-hydroxyvitamin D (25-(OH)D) occurs first in the liver before being converted by the enzyme 1*α*-hydroxylase to the metabolically active form 1*α*, 25-hydroxyvitamin D (25-(OH)2D), primarily in the kidneys [7, 8].

Because of the dependence on UVB light, irrespective of geographical location, vitamin D3 levels are usually found to be lower in the winter and spring when exposure to UVB is likely to be lowest [9].

The way in which vitamin D is synthesized is largely determined by the angle, as a function of latitude, of how the sun's rays make contact with the skin and, as the angle goes over the oblique, the amount produced is reduced and any latitude over 35 degrees sees synthesis decrease significantly because it is outside the optimum wavelength range of 280 to 320 nm [10, 11]. However, research indicates that location, irrespective of latitude, is not a distinctive factor in reported cases of vitamin D3 deficiency. A US study found that latitude, during the months of March to October, was not a reliable factor in predicting insufficiency prevalence. This only becomes relevant during the winter months when the angle of the sun changes [10]. This finding might also correlate with that of an earlier study where the serum concentrations of hospital confined in-patients, indoor workers, and outdoor workers were measured throughout the course of a year and were also compared to the amount of ultraviolet radiation (UV) light produced during the same period [12]. The study revealed that although the UV count was highest in July, the serum D3 concentrations of both the indoor and outdoor workers was highest in October and November, respectively. This indicates that there is a time lag between synthesis and optimal amounts being available. The in-patients however, revealed the highest concentrations during the period of strongest UV, despite the fact that there was no access to natural sunlight.

Although there is a lack of consensus relating to optimal levels of vitamin D, generally serum 25-(OH)D concentrations lower than 50 nmol/L (20 ng/mL) are at least considered to be detrimental to bone health, and levels of insufficiency are established at around <75 nmol/L (30 ng/mL) [13]. However, concentrations in respect of the optimal levels necessary to alleviate potential problems between vitamin D deficiency and many other associated illnesses have not yet been established [14, 15].

Although exposure to UVB is largely responsible for the synthesis of vitamin D, location does not play a major role in identifying who is likely to suffer from a deficiency. As yet, no singular definitive reason has been provided for the growing numbers of sufferers and, considering that people with deficiencies still exist in large numbers in countries where exposure to sunlight of optimal latitude is easily accessible, several other potential causes have been investigated. Although contemporary society has seen a significant decrease in communicable diseases, there has been a considerable increase in noncommunicable chronic ailments in general. In respect of vitamin D3 deficiency specifically, several specific groups have been identified as being more susceptible including the elderly, some institutionalized groups, and pregnant women [14, 16–18].

Aim of this systematic review is to investigate if occupations, and specifically the shiftworking, may be considered as possible contributors to the increased incidence of vitamin D3 deficiency in industrialized nations.

## 2. Materials and Methods

This systematic review was performed according to the Preferred Reporting Items for Systematic Reviews and Meta-Analyses (PRISMA) statement [19]. The research was conducted on the electronic databases PubMed, Scopus, and ISI Web of Knowledge. The combination of terms (“25-hydroxyvitamin D” or “Vitamin d^*∗*^”) AND (“shift work” OR “indoor work”) was used to retrieve studies. The review was conducted in the period between January and April 2018.

### 2.1. Eligibility and Inclusion Criteria and Exclusion Criteria

All the articles, concerning the relationship between shift and indoor work and vitamin D deficiency have been included. No restriction about language or time period was applied. No study design has been excluded. Furthermore, the references of review, letters, comments, editorials, and case reports, identified by the search strategy were checked for retrieving further relevant literature.

### 2.2. Study Selection

The first selection was performed filtering duplicate articles by JabRef 4.10 program The articles identified were selected initially by title and abstract, independently by two researchers, and then each investigator evaluated the inclusion criteria by full text. Disagreements between the two reviewers were resolved by a third one. Articles that take into account relationship between shiftwork, indoor work, and vitamin D3 level were included.

### 2.3. Data Extraction

Data extraction was carried out with the same strategy of the selection of the studies: two researchers collected the data, and the disagreement was resolved by a third researcher. A quality assessment was performed according to the Newcastle-Ottawa Scale (NOS) [20] for observational studies and to Amstar scale [21] for trials. The following characteristics were collected: first author, study design (cross-sectional, systematic review), year of publication, country of the first author, type of work, quality score, and sample size.

## 3. Results

**Figure 1:**
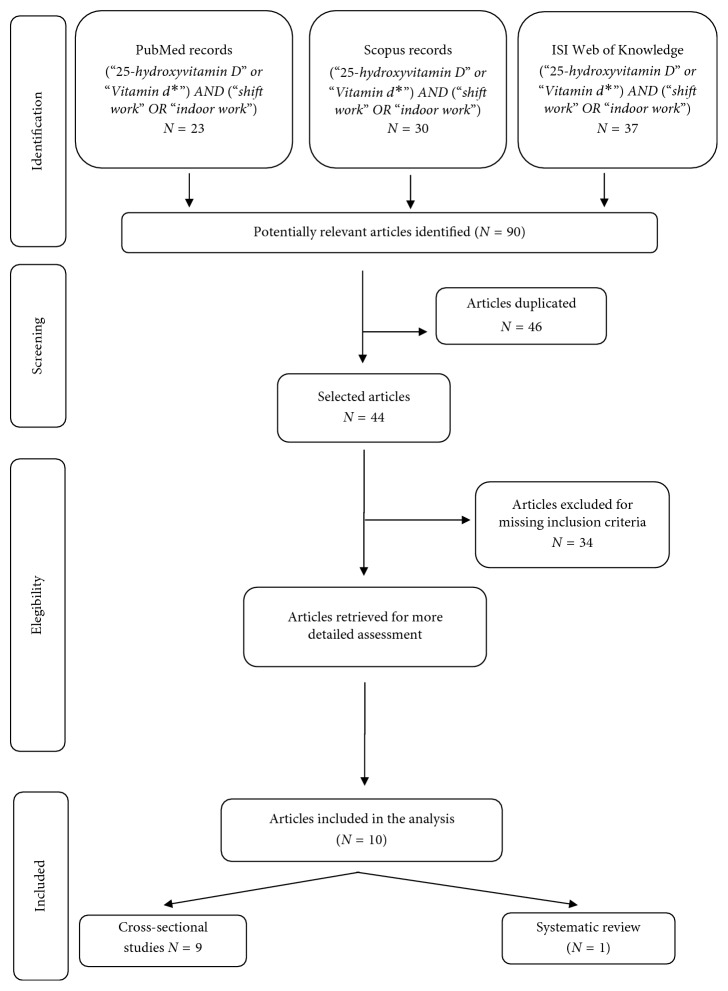
Flow-chart of search strategy.

The selection of articles is shown in the flowchart (Figure 1). Overall 90 papers were found, 23 articles through PubMed, 30 through Scopus, and 37 through ISI Web of Knowledge. Successively, 46 duplicates and 34 articles that did not respect the inclusion criteria were excluded. The remaining papers were analyzed, and from these, 10 articles were finally selected: 9 cross sectional studies [22–30] and 1 systematic review [31].

### 3.1. Characteristics of the Studies

The characteristics of the studies included are shown in Tables 1 and 2.

Cross-sectional study of Ward et al. [22] showed an association between night work and 25(OH)D concentrations in women: 8% lower (95% CI 15% to 2%) in night workers. No association was seen in men, although the author suggested higher mean 25(OH)D in men who worked nights. Two cross-sectional studies of Itoh et al. [23, 24] conducted in Japan in indoor workers found seasonal variation in circulating 25(OH)D and hypovitaminosis D in wintertime in indoor daytime male workers in Japan. Wallingford et al. [25] in a cross-sectional study conducted on shiftwork nurses found 13% of the sample at risk for osteoporosis and/or osteomalacia. Study of Jeong et. al. [26] showed prevalence of vitamin D deficiency among the office workers (88.1%), higher than that of the manufacturing workers (79.0%). Romano et al. [27] evaluates vitamin D status in shiftworkers at an engineering factory in Northern Italy, and 100 male daily workers operating nearby, 25-OH. Vitamin D levels were lower in shiftworkers than daily ones (13.4 ± 5.3 ng/mL versus 21.9 ± 10.7 ng/mL, p50.001). Mizoue et al. [28] found low 25(OH)D concentrations more frequent in young, female, and smokers, engaged in shiftwork and overtime work. Alefishat and Farha [29] evaluated vitamin D status in female night workers that showed significantly lower serum 25(OH)D levels compared to the female day workers (*p*=0.01). The number of night shifts/month was negatively correlated with 25(OH)D levels in both the males and females (*p*=0.01 and *p*=0.007). Lehnert et al. [30] found only small effects of shiftwork in vitamin D levels.

The results of the systematic review of Sowah et al. [31] in which authors calculated the pooled average metabolite level showed that compared to outdoor workers, indoor workers had lower 25-hydroxyvitamin D (25-(OH)D) levels.

## 4. Discussion

Although current research is somewhat limited, the majority of studies strongly indicate that the changing role of labour is at least contributing to the increased prevalence of vitamin D3 deficiency. Despite research suggesting that indoor workers are more commonly susceptible to vitamin D3 deficiency, several occupational groups have been indicated to have lower serum concentrations than others. These include health-care professionals and predominantly, but not exclusively, those where training is involved [32, 33], domestic workers [31], and executives [34]. Numbers of deficient workers within these occupational groups are found to be higher than average even in climates where there is the possibility of high exposure to sunlight.

Unsurprisingly, outdoor workers have been found to be the least likely to suffer from low D3 concentrations; however, when it comes to indoor workers, the highest rate of comparable incidence is often found in those employed on fixed night shift contracts no matter where the location.

### 4.1. Extension of Working Hours

Although globalization has contributed to an extensive transformation not only in the nature of employment but also in respect of working patterns, a European survey (IV) conducted in 2005 concluded that night shift working accounted for 21% of the working population in Europe, and it would seem that this is the occupational group at highest risk of deficiency.

One study compared the concentrations of vitamin D3 in outdoor workers against those of shiftworkers, and the results indicated that only 48% of outdoor workers had levels lower than 50 nmol/L and around 80% of shiftworkers were deficient. However, in some occupational groups, even indoor workers returned results close to that of shiftworkers, with one, hospital trainees, having a 78% incidence levels lower than 50 nmol/L [31].

The changing nature of work then becomes more critical when attempting to analyze predilection for vitamin D deficiency and the groups most likely to be affected. Despite the relatively limited amount of research which has been performed, often it would seem that shiftworkers, irrespective of geographical location, are more predisposed to deficiency than other occupations where fixed daylight hours are the norm [35]. However, the studies would also suggest that a high percentage of workers in specific occupational groups are likely to experience vitamin D deficiency even where the opportunity for exposure to sunlight at the optimal latitude exists.

It should though be noted that a definitive correlation between indoor fixed hour workers and shiftworkers is not always achieved, as this Japanese study investigating differences between serum 25 levels in indoor daytime workers and rotating workers with or without shifts, clearly revealed [23].

Although there is considerable variability among different findings, there is compelling evidence to suggest that shiftworkers and particularly those who work fixed night shift patterns, have a strong predilection toward vitamin D3 deficiency. It is also relevant to examine levels in respect of different seasons to confirm findings. In this study, vitamin D3 levels of workers on fixed night shifts were compared to those who work fixed daytime shifts, not in the summer but in winter when access to ambient UVB was not possible [27].

There are though high number of variables involved in investigating occupational susceptibility to vitamin D3 deficiency which can skew findings, and these include standard practices such as personal supplementation, voluntary supplementation (which exists where manufacturers elect to add vitamin D3 to goods at the production stage and is now common practice), the use of sunscreens which have been strongly promoted particularly in regions where UVB exposure is high, and also the type of work being undertaken which may involve exposure to toxins having the ability to influence vitamin D3 levels. However, once again, studies can often provide paradoxical results. Research undertaken on workers who were exposed to lead and smelting provided conflicting results and in some cases research indicated that those who were exposed to lead on a regular basis could show increased concentrations of vitamin D3 compared to those in different industries [36–40] and were often compatible with average samples taken from the general population [41].

Other factors have also been considered and not least those related to lifestyle issues which are modifiable and include decreased physical activity, unhealthy diets, and use of stimulants such as alcohol, tobacco, and recreational drugs. Social effects, including advanced technology and urbanization may also contribute to the cyclical effect of lifestyle issues. Environmental factors have also been considered to play a role in promoting both mental and chronic conditions generally, and consideration to causation of vitamin D3 deficiency has been given to atmospheric toxins and increased occupational risk factors including stress, which might be induced through physiopathological and psychorelational mechanisms and which are also implicated in many other acute and chronic conditions [19]. However, because vitamin D3 deficiency is now being broadly considered as a possible contributor to many illnesses which were previously unassociated with the condition, the wider implications and corresponding benefits of preventative measures are becoming increasingly relevant.

Shiftworkers are consistently reported as being the occupational group most likely to suffer from a deficiency of vitamin D3. This group has also been investigated in respect of many other health issues including gastrointestinal problems, sleep disorders, and an increased risk of some cancers [41, 42]. Given that vitamin D3 deficiency is now being correlated with other diseases, the imperative to improve levels in shiftworkers is increasing in significance and may also mitigate other, previously unconnected, health problems.

When calculating vitamin D3 deficiency, many factors have to be taken into consideration outside of location. Season, age groups, lifestyle factors, supplementation, gender, and many other facets can play a role. However, averages for vitamin D3 deficiency among general populations appear to start at around 30%. This increases within the working populations, and outdoor workers are the least likely to be affected. In respect of indoor employment, the nature of work plays a role: placement in the hierarchical structure, blue or white collar working, whether pollutants are involved, and the hours of employment. Research is also emerging that suggests the illnesses which can arise, either directly or indirectly, from a deficiency of vitamin D3 are far more extensive than originally believed, and this negatively impacts populations both socially and economically.

The findings are limited by many factors not least the amount of the direct exposure to sunlight relevant to individual participants. Personal supplementation is also rarely established, and the aspect of voluntary supplementation may be impossible to assess simply because participants are often not aware they are eating products with added fortification. However, there can be little doubt that the number of people in employment suffering from a deficiency of vitamin D3 is likely to have negative connotations on a personal and industrial level.

Limits of the study could be the few studies in literature that investigate this phenomenon and the fact that it is not possible to conduct a meta-analysis, because there are few quantitative and heterogeneous data.

It would appear prudent to investigate the potential of providing nutritional education to workers in addition to including preventative measures in the workplace.

## 5. Conclusion

In conclusion, no occupational risk factor has been found to be clearly related to vitamin D3 deficiency.

Although there are many facets associated with investigating deficiencies within occupational groups, there are clear patterns emerging. As would be expected, outdoor workers are less likely than indoor workers to suffer from vitamin D3 deficiency. However, even here, we see that a significant proportion of outdoor workers can, particularly at certain times of year, return serum concentrations which are less than optimal.

Predictably, indoor workers are more likely to experience vitamin D3 insufficiency or deficiency. However, within this broad range, there are segments indicated to have a higher incidence than others, and this can even be higher in specific sectors within the same employment structure even though all employees are restricted from sunlight for the same period of time.

## Figures and Tables

**Table 1:** Characteristics of cross-sectional study.

First author	Country	Year	Study design	Sample	Main results	Type of work	NOS (max 9)
Ward	UK	2011	Cross-sectional study	6134	An association between night work and 25(OH)D concentrations found in women; concentrations 8% lower (95% CI 15% to 2%) in night workers compared with others	Various	7

Itoh	Japan	2011	Cross-sectional study	19	Seasonal variation in circulating 25(OH)D and intact PTH levels, and hypovitaminosis D in wintertime in indoor daytime male workers in Japan	Healthy indoor daytime workers	7

Itoh	Japan	2011	Cross-sectional study	12	Seasonal difference in 25(OH)D between the months of February and October 2008 in indoor daytime workers intact PTH circulating levels were high in February and decreased in October, while no consistent pattern of seasonal variation in 1*α*, 25(OH)2D	Healthy indoor worker	7

Wallingford	USA	2013	Cross-sectional study	83	Most nurses had adequate serum vitamin D status for bone health, 13% were at risk for osteoporosis and/or osteomalacia following winter months	Premenopausal nurses	8

Jeong	Korea	2014	Cross-sectional study	5409	Permanent workers (84.7%) showed a higher prevalence of vitamin D deficiency compared to the temporary workers (78.8%). The prevalence of vitamin D deficiency among the office workers was 88.1%, higher than that of the manufacturing workers (79.0%)	Indoor worker	7

Romano	Italy	2015	Cross-sectional study	196	Shiftworkers had lower levels of vitamin D (Wilcoxon–Mann–Whitney test, *p* < 0.001)	Shift workers at an engineering factory and daily workers operating nearby	7

Mizoue	Japan	2015	Cross-sectional study	1786	Low 25(OH)D concentrations more frequent in young, female, and smokers engaged in shiftwork and overtime work and slept less	Employees of a manufacturing company in the nonferrous metal industry	7

Alefishat	Jordan	2016	Cross-sectional study	140	Female night workers had significantly lower serum 25(OH)D levels compared to the female day workers (*p*=0.01). No significant difference in serum 25(OH)D levels was found between the night and day male workers (*p*=0.25). The number of night shifts/month was negatively correlated with 25(OH)D levels in both the males and females (*p*=0.01 and *p*=0.007)	Night shift worker	8

Lenhert	Germany	2018	Cross-sectional study	67	Lower serum levels were found in samples drawn in winter and spring and in obese subjects. Shiftwork had only small effects on vitamin D levels	Female health care worker	8

**Table 2:** Characteristics of systematic review.

First author	Country	Year	Main result	Amstar score
Sowa	Canada	2017	Compared to outdoor workers, indoor workers had lower 25-hydroxyvitamin D (25-(OH)D) levels (40.6 ± 13.3 vs. 66.7 ± 16.7 nmol/L; *p* < 0.0001). Mean 25-(OH)D levels (in nmol/L) in shiftworkers, lead/smelter workers, and coalminers were 33.8 ± 10.0, 77.8 ± 5.4, and 56.6 ± 28.4, respectively	9/11
